# Gibbosité vertébrale congénitale évoquant un mal de Pott chez un nouveau-né de mère tuberculeuse: à propos d'un cas et revue de la literature

**DOI:** 10.11604/pamj.2014.18.325.5216

**Published:** 2014-08-25

**Authors:** Tina Katamea, Olivier Mukuku, Oscar Numbi Luboya

**Affiliations:** 1 Faculté de Médecine, Université de Lubumbashi, Lubumbashi, République Démocratique du Congo; 2 Ecole de Santé Publique, Université de Lubumbashi, Lubumbashi, République Démocratique du Congo

**Keywords:** Gibbosité vertébrale, nouveau-né, mal de Pott, spinal hump, newborn, Pott disease

## Abstract

Les formes latentes de tuberculose chez la femme enceinte sont associées à un risque élevé de passage à une forme active qui augmente le risque de transmission de la mère infectée à l'enfant dans les 3 premières semaines de vie. Nous rapportons un cas de Gibbosité vertébrale congénitale évoquant un mal de Pott chez un nourrisson de mère tuberculeuse, observé à Lubumbashi, en République Démocratique du Congo.

## Introduction

L'incidence de la tuberculose sur grossesse est croissante dans les pays aux ressources limitées. Dans la plupart de ces pays, cette incidence croissante est associée à l'augmentation de la prévalence de la tuberculose dans la population des femmes en âge de procréer [1]. En Afrique du Sud, par exemple, l'incidence de la tuberculose dans la population féminine a quasiment triplé entre 1991 et 1995, passant de 154/100000 à 413/100000 [2]. En Tanzanie, une étude récente a révélé une prévalence de tuberculose latente de 37,4% chez la femme enceinte [3]. Cette forme latente est associée à un risque élevé de passage à une forme active qui augmente le risque de transmission de la mère infectée à l'enfant de 15% dans les 3 premières semaines de vie [3]. A ce jour, la démarcation entre les formes néonatales acquises de façon congénitale et néonatales acquises en post-natale est encore sujette à controverses [4]. Nous rapportons un cas de Gibbosité vertébrale congénitale évoquant un mal de Pott chez un nouveau-né de mère tuberculeuse, observé à Lubumbashi, en République Démocratique du Congo.

## Patient et observation

Un nourrisson de sexe féminin, âgée de 2 mois, de 50 cm de taille, pesant 2,8 Kg a été admis aux urgences pour fièvre, pleurs incessants, refus de téter et tuméfaction dorsale croissante très marquée à sa troisième semaine de vie. Elle est née à terme à la suite d'une grossesse marquée par une tuberculose pulmonaire à bacilloscopie positive diagnostiquée à 32 semaines d'aménorrhée. Son poids à la naissance était de 2500 grammes (P50). Sa mère est âgée de 34 ans, P5G5A0D0. Sa sérologie VIH est négative. Aucune vaccination par le BCG n'a été rapportée. Depuis l'apparition de la masse, un traitement symptomatique à base de Paracétamol lui a été administré dans un centre de santé de premier échelon.

A l'examen physique, elle était fébrile (38,7°C), tachycarde (150 battements/min), polypnéique (70/min) et sa saturation pulsée en oxygène (SpO2) était de 85% à l'air ambiant. La patiente présentait une pâleur cutanéo-muqueuse et était anictérique. Aucune adénopathie périphérique n’était objectivée dans les aires sous-maxillaires, rétro auriculaires et cervicales. L'examen cardio respiratoire a révélé la présence de fins râles ronflants disséminés dans les deux champs pulmonaires. Son abdomen est légèrement ballonné avec la présence d'une hépatomégalie à 3 cm en dessous du rebord costal, non sensible, à surface régulière et sans reflux hépato-jugulaire. Nous avons par ailleurs noté une splénomégalie (stade 1 selon Hackett).

**Figure 1:**
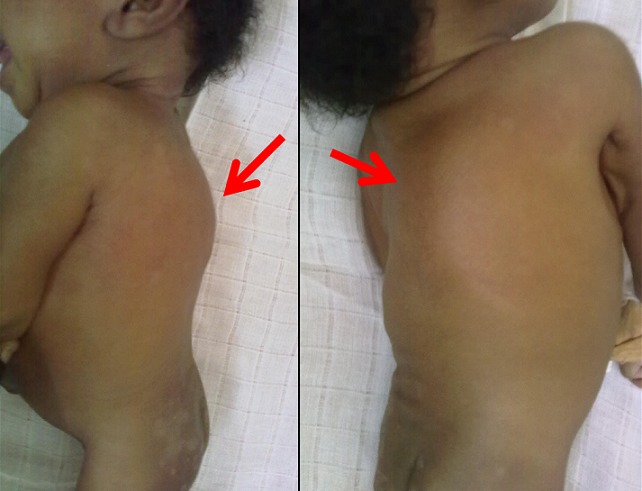
Image montrant la présence d'une gibbosité ainsi qu'une masse arrondie, d'aspect rougeâtre, de surface régulière à hauteur de la colonne dorsale

**Figure 2:**
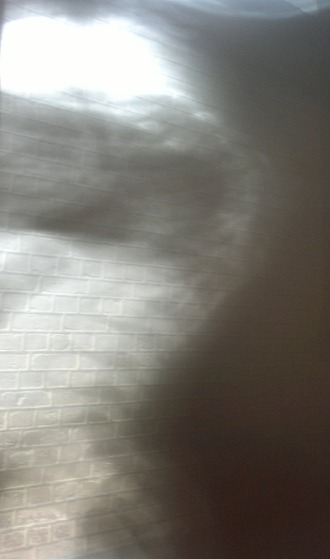
Image radiologique d'incidence de profil mettant en évidence une cyphose secondaire à une destruction et un tassement des vertèbres dorsales

L'examen de la colonne vertébrale révèle la présence d'une gibbosité ainsi qu'une masse arrondie, d'aspect rougeâtre, de surface régulière, de 7 cm de grand diamètre, ferme, sensible et non mobilisable à hauteur de la colonne dorsale (Figure 1). L’évaluation de son état nutritionnel (selon les standards de l'OMS 2006) rapporte une malnutrition sévère (z-score P/A= -4,3 ET, z-score T/A= -3,5 ET et z-score P/T= -2,1 ET). Les examens biologiques montraient une hyperleucocytose (20000 globules blancs/mm3 à prédominance neutrophilique), une vitesse de sédimentation accélérée (80 mm/à la première heure), une protéine C réactive à 102 mg/ml, une anémie (6 mg% d'hémoglobine et 18% d'hématocrite), une élévation des enzymes hépatiques et une PCR- RNA pour le VIH négative. L'intradermo-réaction tuberculinique à H24 d'hospitalisation et lue à H48 positive (10 mm de diamètre d'induration). La radiographie du thorax incidence face était normale. Sur l'incidence de profil, on observait une cyphose secondaire à une destruction et un tassement des vertèbres (Figure 2).

A l'issue de tout ce qui précède, nous avons évoqué le diagnostic de spondylodiscite tuberculeuse néonatale associée à une anémie sévère décompensée. Sa prise en charge était faite d'antipyrétique, d'oxygénothérapie et de transfusion sanguine en urgence. Le décès est survenu à H72 d'hospitalisation, dans un tableau de défaillance multiviscérale, avant l'initiation de la poly-chimiothérapie anti tuberculeuse. Nous n'avons pas obtenu l'autorisation de réaliser une mise en culture des vertèbres lésées pour isoler les bacilles de Koch.

## Discussion

D'après les critères proposés par Beizke en 1935 et révisés de Cantwell en 1994, le diagnostic de tuberculose congénitale impose l'existence de lésions tuberculeuses documentées chez le nouveau-né et au moins un des signes suivants: symptômes survenant dans la première semaine de vie, un complexe d'atteinte hépatique primaire, une tuberculose génitale maternelle ou placentaire et l'exclusion d'une contamination post-natale [5]. La démarcation entre les formes néonatales acquises de façon congénitale et néonatales acquises en post-natale est encore difficile à établir en pratique clinique et ne comporte qu'un intérêt épidémiologique.

La transmission se fait par voie hématogène à travers la veine ombilicale avec des lésions primaires hépatiques ou à la suite de l'inhalation ou de l'ingestion du liquide amniotique infecté [6]. La présentation clinique de la tuberculose congénitale n'est pas très spécifique. Ceci rend le diagnostic particulièrement difficile dans cette population pédiatrique. En effet, la détresse respiratoire, la léthargie, le refus de s'alimenter, la fièvre, l'irritabilité, ainsi que l'hépatosplénomégalie peuvent être aussi retrouvés dans les sepsis bactériens, les infections néonatales à Herpès virus,‘ De plus, chez le nouveau-né et les nourrissons de moins de 6 semaines, l'immaturité du système immunitaire rend aléatoire l'intradermoréaction à la tuberculine. Dans le cas de notre patient, une induration d'environ 10 millimètres de diamètre a été observée à H48 d'hospitalisation, la tuméfaction dorsale présente dès la naissance, son caractère inflammatoire et rapidement progressif dans les premières semaines de vie, l'augmentation des transaminases d'une part, ainsi que la tuberculose pulmonaire à bacilloscopie positive non traitée documentée pendant la grossesse chez la mère d'autre part, constituent des arguments en faveur d'un mal de Pott congénitale chez ce nourrisson. Les parents n'ont pas autorisé la mise en culture en post-mortem des vertèbres dorsales lésées. Cet examen aurait pu nous permettre de documenter formellement l'infection tuberculeuse.

La cyphose congénitale est une déformation vertébrale dans le plan sagittal résultant d'une flexion excessive de la région spinale concernée. Ce type de malformation peut être classé en défaut de formation, de segmentation ou dislocation spinale et leur cause est à ce jour considérée comme multifactorielle. Des facteurs génétiques et environnementaux tel que le diabète maternel ainsi que les médicaments anticonvulsivants ont été associés à ces malformations [7]. Ici, les défauts de la partie antérieure des corps vertébraux ainsi que la croissance de la partie postérieure « saine » est responsable du caractère évolutif de la malformation. Dans les cas graves de mal alignements du canal médullaire, une paraplégie peut être observée à la naissance [8]. Le spina bifida qui résulte d'un défaut de fusion des arcs vertébraux embryonnaires peut se localiser à n'importe quelle hauteur du rachis [8]. Une tuméfaction sous-cutanée peut être observée au niveau de la colonne dorsale si le défect vertébral se situe à cette hauteur. Dans ces cas, des anomalies cutanées en regard de la masse sont généralement observées (pilosité anormale, irrégularité cutanée) [8]. Dans le cas de notre patient, il n'y avait aucune anomalie de ce type. Par ailleurs, la radiographie de la colonne n'a révélé aucun défect type défaut de soudure postérieure des arcs vertébraux.

Dans le cas de notre patient, aucun traitement antituberculeux n'a été administré à la mère pendant la grossesse. Pourtant, les indications de la chimiothérapie antituberculeuse chez la femme enceinte ne sont pas différentes de celles chez la femme non enceinte [9]. Une bithérapie est instaurée aux doses usuelles et la durée du traitement n'est pas influencée par la grossesse de la patiente. Bien que traversant la barrière placentaire, aucun effet tératogène n'a à ce jour été associé à l'isoniaside et la pyrazinamide [10]. La rifampicine devrait cependant être utilisée prudemment du fait de sa capacité d'inhibition de l'ARN polymérase. S'agissant de l'ethambuthol, 2,2% de malformations f'tales ont été rapportées dans une série de 638 nouveau-nés dont les mères étaient traitées par l'ethambutol durant la grossesse. La streptomycine a été associée à un risque accru d'ototoxicité et de surdité; d'où la recommandation de la proscrire pendant la grossesse. Dans tous les cas, ces grossesses à risque devraient être suivies dans des hôpitaux de référence plutôt que dans les centres de santé de niveau 1. Les nouveau-nés présentant une tuberculose périnatale (congénitale ou néonatale) devraient recevoir isoniaside (10-30 mg/kg/j), rifampicine (10-20 mg/kg/j), pyrasinamide (15-30 mg/kg/j) et streptomycine (20-30 mg/kg/j) ou ethambutol (15-25 mg/kg/j) pour les 2 premiers mois, suivi d'un traitement par isoniaside et rifampicine pour 4-10 mois en fonction de la sévérité du type d'atteinte tuberculeuse [5].

Dans le premier cas clinique présentant une tuberculose vertébrale rapport dans la littérature par Grover en 2003, le diagnostic précoce suivi d'une poly chimiothérapie a permis d'obtenir une guérison complète [11]. En dehors de ce cas heureux, la tuberculose du nouveau-né est grevée d'une mortalité proche de 50% [12]. La tuberculose vertébrale de l′enfant demeure, à l'heure actuelle, grave car rapidement évolutive et extensive [13, 14].

## Conclusion

Lorsque la tuberculose maternelle n'est pas traitée et le diagnostic retardé chez l'enfant, le pronostic est fatal et le décès peut survenir à la suite d'un sepsis foudroyant.
